# Early failure for wear after ceramic-on-highly cross-linked polyethylene total hip arthroplasty: a case report

**DOI:** 10.1186/s12891-020-03697-1

**Published:** 2020-10-10

**Authors:** Chenkai Li, Haining Zhang

**Affiliations:** Department of Joint Surgery, the Affiliated Hospital of Qingdao University, Qingdao, 266000 Shandong China

**Keywords:** Dysplasia dysfunction of the hip, Ceramic-on-highly cross-linked polyethylene, Total hip arthroplasty, Case report

## Abstract

**Background:**

Highly cross-linked polyethylene (HXLPE) enhances the anti-wear characteristics of the conventional polyethylene (PE). Early failure for wear after ceramic-on-highly cross-linked polyethylene (CoHXLPE) total hip arthroplasty (THA) is extremely rare.

**Case presentation:**

We described the case of a 60-year-old man who underwent right CoHXLPE THA because of the developmental dysplasia hip (DDH) complained pain 32 months after this procedure. Plain radiographs showed that eccentric wear existed at the polyethylene insert. However, the patient refused surgery at that time and did not stop weight-bearing. The right hip pain continued for 7 months. Plain radiographs of the pelvis showed that the HXLPE liner was penetrated and partial inner wall of acetabular shell was worn. Acetabular cup revision was performed, and the ceramic head and HXLPE were exchanged.

**Conclusions:**

Difficult reduction during primary THA, especially for DDH, can result in higher abductor tension, which may lead to early eccentric wear of the prosthesis. Whenever eccentric wear of HXLPE liner was found, weight-bearing must be stopped to avoid the accelerated wear and adverse reactions to metal debris (ARMD).

## Background

THA is an effective treatment for various end-stage hip diseases, such as osteonecrosis, rheumatoid arthritis, degenerative joint diseases and degenerative diseases caused by developmental dysplasia [1]. Nowdays, because of the improved bearing surfaces and materials, THA lasts longer than before [2]. However, even with these enhancements, failure caused by polyethylene wear, instability, aseptic loosening, malpositioning, and infection is inevitable.

Wear debris production from bearing surfaces is regarded as the main factor limiting THA survival. In order to improve the anti-wear characteristics of the conventional PE, HXLPE has been developed to reduce the amount of wear particle production. These improved PEs have shown better wear characteristics in vitro and in vivo [3]. Although it is rare, we found a case of early failure of CoHXLPE bearing only 32 months after THA.

## Case presentation

**Fig. 1:**
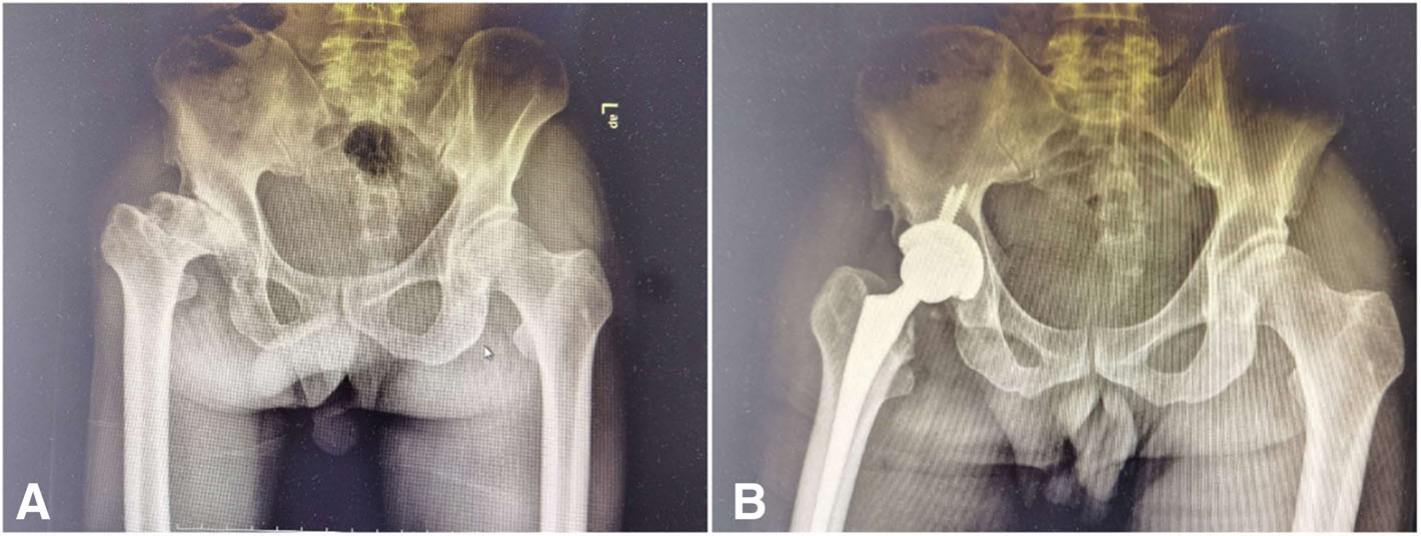
DDH was found at the right hip before surgery (**a**), and the size and position of the prothesis was good 1 week after THA (**b**)

**Fig. 2:**
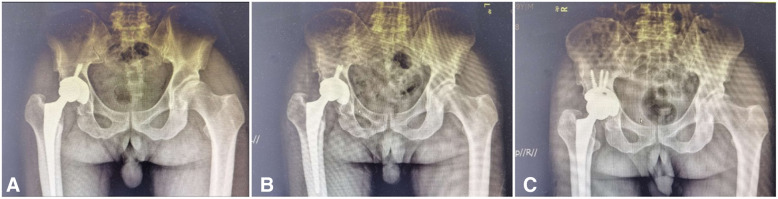
Plain radiographs at 32 months (**a**) showed initial eccentric wear of the prosthesis. Seven months later, the HXLPE was penetrated and partial inner wall of acetabular shell was worn (**b**). Plain radiographs of the pelvis showed ideal size and position of the prosthesis (**c**)

A 60-year-old man diagnosed with developmental dysplasia of the right hip (Fig. 1a) underwent CoHXLPE THA through lateral approach (Fig. 1b) at local hospital in 2017. The patient’s first THA as performed by a sophisticated surgeon, although he was admitted in a local hospital. The BMI of this male patient was 22. The DDH classification of the patient was Crowe III, as it can be seen in Fig. 1. The operation time was 75 min, and blood loss was 260 ml. A 50 mm porous coated shell, HXLPE liner, 36 mm ceramic head and fully coated stem were inserted. No osteotomy was needed for reduction of the hip. No complications during and after surgery were found. The reduction of hip joint was slightly tense during primary THA, but the original outcomes were satisfied. However, after 32 months, he reported weight-bearing pain in his right hip with no obvious reasons. Plain radiographs of the pelvis showed that eccentric wear existed at the superolateral part of the prosthesis (Fig. 2a). The patient hesitated to receive surgical treatment and, meanwhile, he did not stop weight-bearing. The right hip pain continued for 7 months. Plain radiographs and CT scan of the pelvis showed accelerated wear and large amounts of metal particles, of which HXLPE liner was penetrated and partial inner wall of acetabular shell was worn (Fig. 2b).

**Fig. 3:**
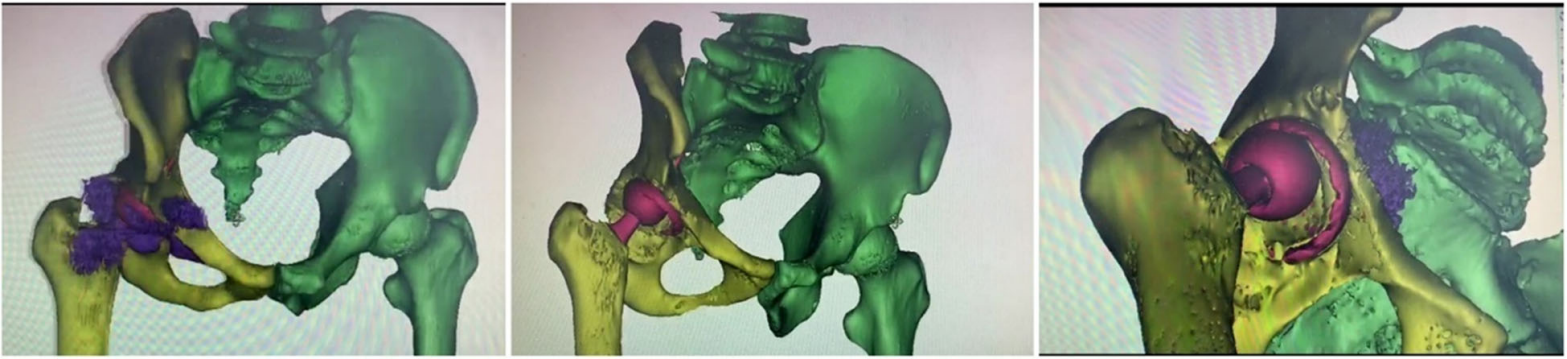
3D computed tomography reconstruction revealed numerous metal debris (purple) around hip joint and worn of the ceramic head and acetabular shell

**Fig. 4:**
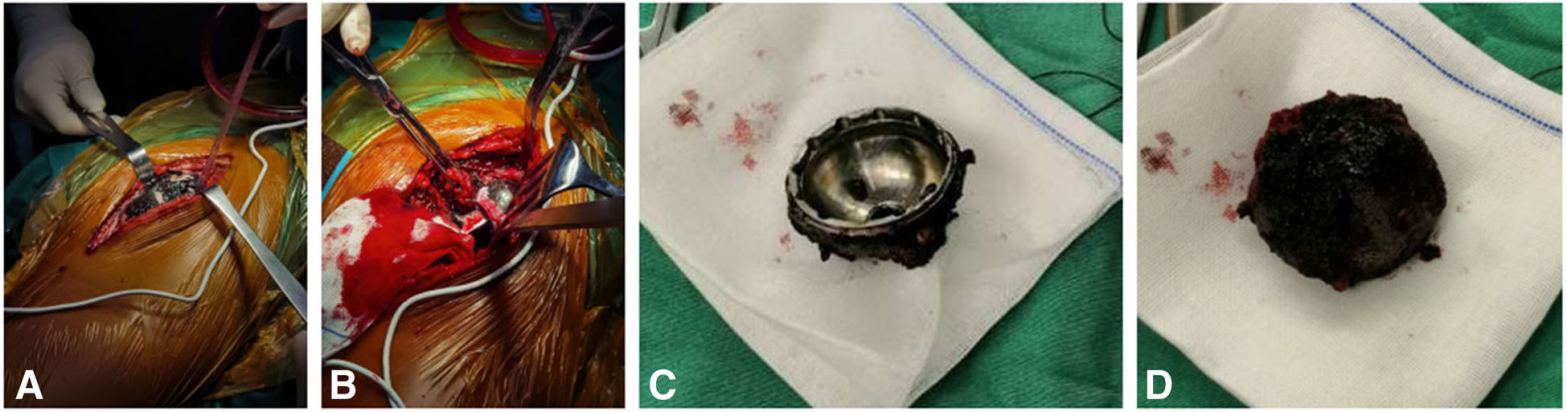
Metal debris were distributed in the articular cavity, abductor and fascia lata (**a**), as well as the ceramic head (**b**). The superolateral part of the inner wall of acetabular shell became thin (**c**). Good bone ingrowth can be seen at the back of the cup (**d**)

Infection was excluded before revision, as the white blood cell count, C- reactive protein level and erythrocyte sedimentation rate were negative. Metal ion concentrations in the blood were not measured because this was not covered by his health insurance. 3D computed tomography reconstruction revealed numerous metal debris around hip joint, and the worn between ceramic head and the acetabular shell (Fig. 3). Intraoperatively, large amounts of metal debris distributed within the joint cavity, abductor and fascia lata (Fig. 4a). Superolateral part of the HXLPE liner was penetrated, loosen and migrated. Surface of the ceramic head was also covered by metal debris (Fig. 4b). The inner wall of acetabular shell became thin due to wear (Fig. 4c), however, it was well-fixed. The stem was stable to be left in situ. Good bone ingrowth was found at the back of the cup (Fig. 4d) Metal debris migrated into the bottom of the acetabulum through the screw holes. Excessive debridement of granulomatous tissue and metal debris was performed. Two millimeter larger shell was inserted without bone graft, and three screws were used to enhance the primary stability. Ceramic head and HXLPE liner were also exchanged. Intravenous antibiotics was used for 3 days after operation. A drainage tube was inserted and removed 24 h after surgery.

The patient was permitted partial weight-bearing 3 days after surgery, and fully weight-bearing after 4 weeks. The muscle strength recovered 4 weeks after operation. The anteversion angle, abduction angle were all within normal range. Plain radiographs of the pelvis showed ideal size and position of the prosthesis (Fig. 2c). Pain was relieved at 3 weeks follow-up, and the patient returned to normal life 8 weeks postoperatively.

## Discussion and conclusions

HXLPE was developed to reduce wear in conventional PE liners. Meanwhile, the incidence and severity of osteolysis secondary to wear would also be decreased [4]. Mutimer et al. [5] reported that HXLPE had a lower revision rate compared with traditional PE with an average follow-up period of 5.5 years. In addition, the use of ceramic heads reduces wear rates as well [6]. The reported wear rate of CoHXLPE was 0.04–0.20 mm per year [7]. But in the current case, the HXLPE was penetrated only 3 years after primary THA, which led to further wear between ceramic head and acetabular shell. To the author’s knowledge, this is the first report of early failure for wear after CoHXLPE THA.

The wear of HXLPE after THA is multifactorial, such as body weight, high activity level, large abduction angle of the cup and decrease of femoral offset [8–10]. What’s more, DDH is also related to the wear of HXLPE. In Crowe III and IV DDH, soft tissue around the hip contractures because the femoral head is chronically dislocated. When the rotation center moves down or returns to the true acetabulum, the tension of the soft tissue may become much higher, which may accelerate the wear of HXLPE [11]. In the current case, the patient’s DDH classification was Crowe III and failure mechanism may be ascribed to the high tension of abductor after reduction of DDH, which eventually led to the rapidly superolateral eccentric wear of the HXLPE.

With the image test, the early wear of the prosthesis can be found. Wear debris production may lead to ARMD, which will worsen the fibrosis and necrosis of soft tissue, the erosion of metal and the loosening of prosthesis [12]. Thus, once eccentric wear occurs, the patient must stop weight-bearing to prevent the accelerated wear and osteolysis.

Revision THA is the definitive treatment for the wear of PE [13]. However, whether a well-fixed acetabular cup should be revised or not, there is still no general consensus or specific guideline. Restrepo et al. [14] advocated that the acetabular component should be revised when the femoral head penetrated the liner and damaged the metal cup, or the locking mechanism was damaged, or the component was malpositioned which may lead to the instability of the revision. Besides, Maloney et al. [15–17] divided the uncemented cups into three types: I. The cup was radiographically stable (following six principles must be met: (1) the implant should be a modular implant, (2) the implant should have an acceptable track record, (3) the cup was in good position, (4) the locking mechanism could not be damaged, (5) the metal shell was intact, and (6) the thickness of the polyethylene liner replacement must be adequat.); II. The cup was radiographically stable (the shell was radiographically stable, but any of the previous principles were not met.); III. The cup was radiographically unstable. As to type I, a liner exchange can be performed. However, component should be revised in type II and type III. Besides, graft may or may not be used in all types. However, even with perfect preoperative assessment, it should be noted that intraoperative testing of component positioning and stability is also essential. In the current patient, although the cup was well-fixed, it had been worn inside. Thus, we performed acetabular revision and ceramic femoral head and HXLPE exchange. The revision obtained satisfactory early outcome.

Difficult reduction during primary THA, especially for DDH, can result in higher abductor tension, which may lead to early eccentric wear of the prosthesis. Whenever eccentric wear of HXLPE liner was found, weight-bearing must be stopped to avoid the accelerated wear and ARMD. Partial or total revision THA is the primary treatment for such wear of HXLPE. A careful assessment of preoperative radiographs and intraoperative testing are imperative to avoid subsequent dislocation and loosening.

## Data Availability

The final dataset will be available from the corresponding author.

## Abbreviations

HXLPE: highly cross-linked polyethylene
PE: polyethylene
CoHXLPE: ceramic-on-highly cross-linked polyethylene
THA: total hip arthroplasty
DDH: developmental dysplasia hip
ARMD: adverse reactions to metal debris
